# Geochemical characterization of oceanic basalts using Artificial Neural Network

**DOI:** 10.1186/1467-4866-10-13

**Published:** 2009-12-23

**Authors:** Pranab Das, Sridhar D Iyer

**Affiliations:** 1 National Institute of Oceanography (Council of Scientific & Industrial Research) Dona Paula Goa 403004, India

## Abstract

The geochemical discriminate diagrams help to distinguish the volcanics recovered from different tectonic settings but these diagrams tend to group the ocean floor basalts (OFB) under one class i.e., as mid-oceanic ridge basalts (MORB). Hence, a method is specifically needed to identify the OFB as normal (N-MORB), enriched (E-MORB) and ocean island basalts (OIB).

We have applied Artificial Neural Network (ANN) technique as a supervised Learning Vector Quantisation (LVQ) to identify the inherent geochemical signatures present in the Central Indian Ocean Basin (CIOB) basalts. A range of N-MORB, E-MORB and OIB dataset was used for training and testing of the network. Although the identification of the characters as N-MORB, E-MORB and OIB is completely dependent upon the training data set for the LVQ, but to a significant extent this method is found to be successful in identifying the characters within the CIOB basalts. The study helped to geochemically delineate the CIOB basalts as N-MORB with perceptible imprints of E-MORB and OIB characteristics in the form of moderately enriched rare earth and incompatible elements. Apart from the fact that the magmatic processes are difficult to be deciphered, the architecture performs satisfactorily.

## Introduction

Several discrimination diagrams have been proposed to classify the ocean floor basalts (OFB) into ocean island basalts (OIB), mid-oceanic ridge basalts (MORB), and island arc basalts (IAB) that are recovered from different tectonic settings. These diagrams are constructed by considering a variety of oxides and/or their ratios, for instance the triangular diagrams of Ti/100 - Zr - Y.3 by Pearce & Cann [1], Hf/3 - Th - Ta by Wood et al. [2], TiO_2 _- MnO - P_2_O_5 _× 100 by Mullen [3] and 2Nb -Zr/4 -Y by Meschede [4]. The model based geochemical studies classify the MORB into three types such as normal - , enriched or plume - and transitional MORB (i.e., N- MORB, E or P-MORB and T-MORB, respectively) or as OIB [5-7]. The discrimination diagrams provide a broad picture of the type of basalts but it is difficult to determine the basic characters that are involved in the geochemical classification of OFB based solely on the above mentioned elements and oxides. Recently, Sheth [8] considered several log-ratio and discriminant-analysis based diagrams to evaluate and classify the basalts into OIB, island arc basalts (IAB) and MORB. The suggested discriminate diagrams helped to distinguish the volcanics recovered from different tectonic settings but group the OFB under one class i.e., as MORB. Hence, a method is needed to specifically identify the OFB as N-MORB, E/P-MORB and OIB.

Therefore, other than through conventional discrimination plots, a methodology is explored for an improved technique to characterise and evaluate the various basaltic characters in a geochemical dataset. We found that a hybrid Artificial Neural Network (ANN) architecture, also known as Learning Vector Quantisation (LVQ) which is a supervised network, could better help to characterise the OFB. As a supervised method, LVQ uses known target output classifications for each input pattern of the form. Some instances where LVQ architecture has being extensively used are for pattern recognition and seafloor classification [9] and characterisation of the seafloor sediments [10]. In this communication we use the LVQ approach in order to determine the inherent geochemical characters and to classify the Central Indian Ocean Basin (CIOB) basalts.

## Learning Vector Quantisation (LVQ) Architecture

An ANN is an information processing paradigm that is inspired by the way biological nervous systems, such as the brain, process information. The key element of this pattern is the novel structure which is composed of a large number of highly interconnected processing elements (neurons) working in unison to solve specific problems. An ANN is configured for a specific application, such as pattern recognition or data classification, through a learning process. Learning in biological systems involves adjustments to the synaptic connections that exist between the neurons. This is true of ANNs as well.

**Figure 1:**
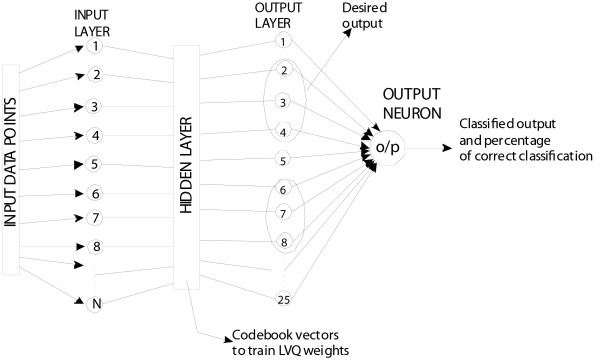
**LVQ architecture to show one hidden layer with Kohonen neurons, adjustable weights between input and hidden layer and a winner takes it all mechanism**.

LVQ constitutes a powerful and intuitive method for adaptive nearest prototype classification. The LVQ architecture is based on the weight-updating rule to obtain the characteristics of the learning data. In a feed-forward ANN (Fig. 1) the data travel one way" from input to output with no feedback (loops) i.e., the output of any layer does not affect that same layer. Feed-forward ANN tends to be a straight forward network that associates inputs with outputs. LVQ algorithms do not approximate density functions of class samples as is the case for Vector Quantisation or Probabilistic Neural Networks, but directly define the class boundaries based on prototypes, a nearest-neighbour rule and a 'winner-takes-it-all' paradigm [11]. The LVQ is an algorithm for learning classifiers from labeled data samples. Instead of modeling the class densities, it models the discrimination function defined by the set of labeled codebook vectors (CVs) and the nearest neighbourhood search between the codebook and data. During classification, a data point xi is assigned to a class according to the class label of the closest CV. The training algorithm involves an iterative gradient update of the winner unit. The direction of the gradient update depends on the correctness of the classification using a nearest neighbourhood rule in Euclidean space. If a datum sample is correctly classified (i.e., the labels of the winner unit and the sample are the same), the model vector closest to the sample is attracted towards the sample; if incorrectly classified, the sample has a repulsive effect on the model vector.

The objective of LVQ is to cover the input space of samples with CVs, each representing a region labeled with a class. A CV can be considered as a prototype of a class member, localized in the center of a class or decision region in the input space. A class can be represented by an arbitrary number of CVs, but one CV represents one class only. In terms of neural networks a LVQ is a feed-forward net with one hidden layer of neurons, fully connected with the input layer. A CV can be seen as a hidden neuron ('Kohonen neuron') [11] or a weight vector of the weights between all input neurons and the concerned Kohonen neuron [12], respectively (Fig. 1). Here 'weights' refer to the value of the individual vector in the matrix. In contrast to the standard LVQ, where the winner unit (neuron) is defined with a nearest-neighbour rule in the Euclidean space, we now have a winner unit which minimizes the negative log likelihood of the data. Equivalently, this maximum likelihood unit m_c _is defined by:(1)

where θ_k _is the weight of CVs.

'Learning' means modifying the value of CVs in accordance with adapting rules [11] and therefore, changing the position of a CV in the input space. Since class boundaries are built piecewise - linear segments of the mid-planes between CVs of neighboring classes - these are adjusted during the learning process. The tessellation (a tessellation or tiling) of the plane is a collection of figures that fills the plane with no overlaps and no gaps) induced by the set of CVs is optimal if all data within one cell indeed belong to the same class. Classification after learning is based on a presented sample's vicinity to the CVs. The classifier assigns the same class label i.e., the label of the cell's prototype (the CV nearest to the sample) to all the samples that fall into the same tessellation.

The core of the heuristics [11] is based on a distance function - usually the Euclidean distance is used - for comparison between an input vector and the class representatives. The Euclidean distance [d(i)] is calculated by the equation:(2)

This distance expresses the degree of similarity between presented input vector and CVs. A shorter distance corresponds to a high degree of similarity and a higher probability for the presented vector to be a member of the class represented by the nearest CV. Therefore, the definition of class boundaries by LVQ is strongly dependent on the distance function, the start positions of CVs, their adjustment rules and the pre-selection of distinctive input features. The CV update equation during learning phase, as defined by the nearest-neighbour rule, and a datum sample x(t) are fed in the equation 3 to change the CVs(3)

where the sign depends on whether the datum sample is correctly classified (+) or misclassified (-). The learning rate α(t) ∈ [0, 1] decreases monotonically with time. For different picks of data samples from our training set, this procedure is repeated iteratively until a convergence occurs. Kohonen12 also presents optimized learning-rate LVQ, where the learning-rate is individually optimized for each codebook. The learning function (α) for LVQ1[10-12] uses small values and was optimized to: for right (0.1/t^0.1^) and wrong (0.1/t^0.06^) classifications. During the training and testing of LVQ1, the randomly generated weight matrix was tuned for a particular character in the data set. The LVQ1 network learns all the possible variations for a particular data set and in order to obtain the optimum iteration, we continuously changed the number of iteration steps from a small number to a large one with continuous observation of classification of the data. It was noticed that irrespective of the number of neurons, 30 iterations were optimum for classifying the CIOB basalts.

The basic LVQ algorithm i.e., LVQ1 rewards correct classifications by moving the CV towards a presented input vector, whereas incorrect classifications are punished by moving the CV in an opposite direction. The magnitudes of these weight adjustments are controlled by a learning rate [11] which can be lowered over time so as to acquire finer movements in a later learning phase. Improved versions of LVQ1 are Kohonen's OLVQ1 (with different learning rates for each CV in order to obtain a faster convergence) and LVQ2, LVQ2.1 and LVQ3. Since LVQ1 tends to push CVs away from decision surfaces, it can be expected to search for a better approximation by adjustments of two CVs belonging to adjacent classes. Therefore, in LVQ2 adaptation occurs only in regions with a few cases of mis-classification in order to achieve finer and better class boundaries. While LVQ2 allows adaptation for correctly classifying CVs, LVQ3 leads to an even more weight adjusting operations due to less restrictive adaptation rules.

The accuracy of classification and, therefore generalization and the speed of learning depends on several factors. Generally, the developer of a LVQ has to prepare a learning schedule and a plan as to which LVQ-algorithm(s) - LVQ1, OLVQ, LVQ2.1 etc. - should be used with values for the main parametres during the different training phases. Also, the number of CVs for each class must be decided in order to reach an high classification accuracy and generalization while avoiding under- or over-fitting of the CVs. Additionally, the rules for stopping the learning process as well as the initialization method (e.g., random values, values of randomly selected samples) determine the results.

In this study we have implemented the LVQ1 network to classify the CIOB basalts without placing emphasis on the geographical locations of the samples. The LVQ1 algorithm is such that if the class levels of the input and closest matching reconstruction vectors are the same, then the weights are moved closer to the input vector. Conversely, a mismatch between the two causes the weight to move away from the input vector. This concept is termed as "reward-punishment". Randomly generated weight matrix is used as an initial weight distribution for LVQ1. The weight update equations are implemented on the winning neuron for each input vector presented, with alternate testing and training throughout the dataset. The weight updating takes place following the above equation #3.

The LVQ1 was used as a single layer for classification of the CIOB basalts and thirty five samples were used to train the network with every sample containing twenty one variables. The LVQ1 testing was carried out on known and classified basalt data set [13-21] so as to optimize the weight matrix and to store the characters of the training data. Optimization is a basic step that helps the network to classify the unknown basalt data.

To improve the performance of the LVQ1, it was found that an output neuron grid size of 25 × 1 which represents the different class is most favorable for this study. An increase in the number of neurons would lead to more time to perform a specified work (Table 1). If the number of classified group increases then to avoid the overlap, the number of neurons can be increased and LVQ2 and LVQ3 can be implemented to strengthen the classification. The LVQ1 architecture was written using Matlab 6.1 and the program was run on a P4 (1.70 GHz) computer with 256 MB RAM.

**Table 1:** Time taken by the computer to identify the basalt characters while using different number of output neurons.

	25 Neuron	50 Neuron	75 Neuron
Time lapsed (sec) →			
**N-MORB**	0.4530	0.6100	1.6720
**E-MORB**	0.4220	0.5400	0.5630
**OIB**	0.4690	0.6410	0.7500

## Use of LVQ to Classify Oceanic Basalts

As stated earlier, based on geochemical data the OFB have been classified as N-, E/P- or T-MORB or OIB. Recently, Lacassie et al. [22] have used self organizing map (SOM) based ANN to classify the volcanic rocks. But it is difficult to determine the inherent geochemical characters of the samples with respect to N-MORB, E/P-MORB and OIB, until and unless the network has pre-defined parametres to separate the geochemical characters of the data. Therefore, an attempt is made to introduce the LVQ method for classification and to unravel discrete geochemical traits of the OFB by using certain characteristic elemental concentration of these basalts. In order to classify the OFB we considered one major oxide (K_2_O), seven trace (Sc, Rb, Sr, Y, Zr, Nb and Ba,), six rare earth elements (REE) (La, Ce, Nd, Sm, Eu, and Yb) and seven elemental ratios (Zr/Nb, Y/Nb, Ba/Nb, Zr/Y, Sm/Nd, La/Yb and Ce/Y). A reason for utilizing the above mentioned elements and their ratios is because these carry the geochemical signatures of the individual OFB type i.e., N-MORB, E/P-MORB and OIB [7]. A criterion that we considered while selecting the samples for training and testing, was that the data should not be solely from one sampled site in the CIOB.

K_2_O is a major oxide, which varies systematically in N-MORB, E/P-MORB and OIB. The trace elements (Nb, Yb) and REE (La, Ce, Sm and Eu), signify the characters of the MORB and their ratios typify the different MORB. The incompatible elements (Zr, Rb, Sr, Ba) characterize the MORB as well as provide significant information regarding the nature of the source and magmatic processes. A systematic increase of incompatible elements can be seen from N-MORB to OIB (Table 2). The variation in a few elements could suggest a combination of geochemical makeup of the MORB and this can be deciphered by using the LVQ method.

**Table 2:** The representative MORB values used in this study

	N-MORB		E-MORB		OIB	
	1	2	3	4	5	6
K_2_O	0.04	0.11	1.18	0.63	1.28	0.91
Sc	39	46	45.4	40.3	29.5	26.6
Rb	0.47	1.22	31	14	25.9	12.67
Sr	96	103	433	283	848	1119
Y	24	35	32	25	18.75	16
Zr	55	89	222	98	46.21	31.6
Nb	1.06	2.16	17	13	0.95	0.77
Ba	6.06	14.9	375	201	241	429
La	1.99	3.44	22.5	11.3	9	10.64
Ce	6.11	9.64	50.7	23.7	18.79	22.15
Nd	5.96	8.92	25.6	13	13.51	15.15
Sm	2.18	3.10	6.09	3.32	3.54	3.67
Eu	0.81	1.21	2.23	1.13	1.24	1.27
Yb	2.42	3.43	2.81	2.45	1.68	1.34
Zr/Nb	51.8	41.4	13.06	7.54	48.64	41.04
Y/Nb	22.4	16.0	1.88	1.92	19.74	20.78
Ba/Nb	5.74	6.90	22.06	15.46	253.68	557.14
Zr/Y	2.31	2.58	6.94	3.92	2.46	1.98
Sm/Nd	0.37	0.35	0.24	0.26	0.26	0.24
La/Yb	0.82	1	8.01	4.61	5.36	7.94
Ce/Y	0.25	0.28	1.58	0.95	1	1.38

1-2 le Roux et al. [20]

3-4 Humphris & Thompson [15]

5-6 Raos & Crawford [21]

**Figure 2:**
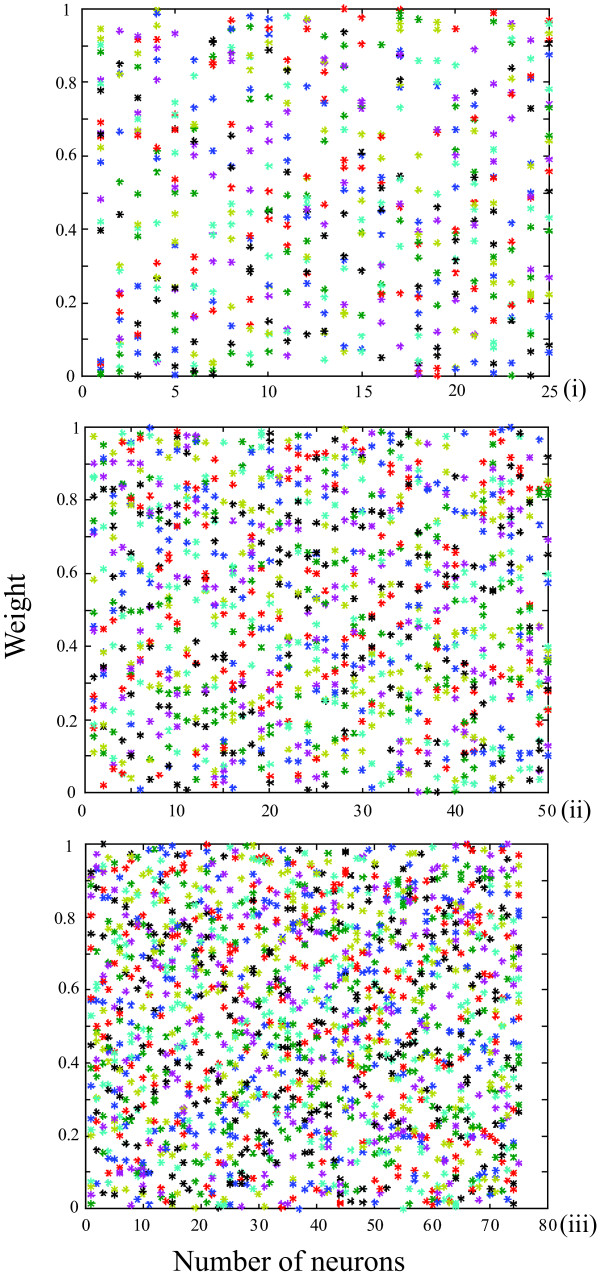
**Initial weight matrix of CVs for LVQ architecture of (i) for 25 neurons (ii) for 50 neurons and (iii) for 75 neurons respectively**.

**Figure 3:**
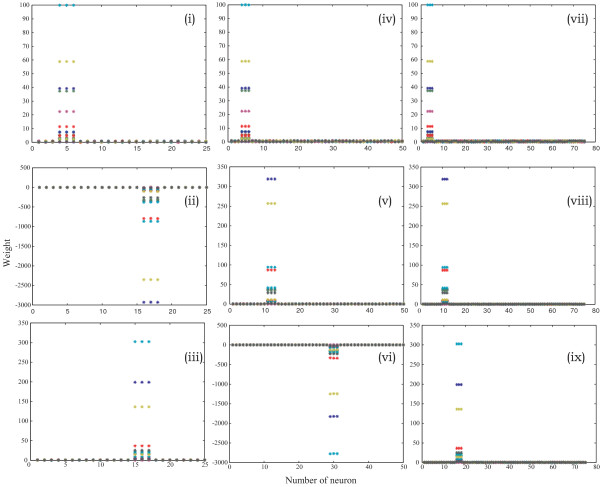
**Final weight matrix of CVs for 25, 50 and 75 neurons after successful training of the system, respectively**. (i), (iv) and (vii) represent the trained CV for N-MORB (ii), (v) and (viii) represent the trained CV for E-MORB (iii), (vi) and (ix) represent the trained CV for OIB

The initial matrix of CVs of unbiased random number of 25 × 21, 50 × 21 and 75 × 21 (Fig. 2) was generated, saved and subsequently used for training of LVQ1 architecture prior to classification. Figure 3 represents the trained weight matrix of 25, 50 and 75 neurons for N-MORB, E-MORB and OIB respectively. To use the LVQ technique, a separate data set for training and testing was arranged in a 21 × n pattern for N-MORB, E/P-MORB and OIB. Here '21' represents the properties of the basalts in terms of the elements and their ratios and 'n' indicates the number of data strips used in the study. The data which were previously classified as N-MORB, E/P-MORB and OIB using classical geochemical criteria, were selected for the study and at the same time the previously defined data were divided in to two sets, one set for training of the network and the other set to observe the performance of the network. During training of the network, the CVs get updated and classify the basalts into the different categories. From the network of 25 neurons we selected output neurons 4 to 6, 10 to 12 and 16 to 18 to designate the N-MORB, E/P-MORB and OIB respectively.

**Figure 4:**
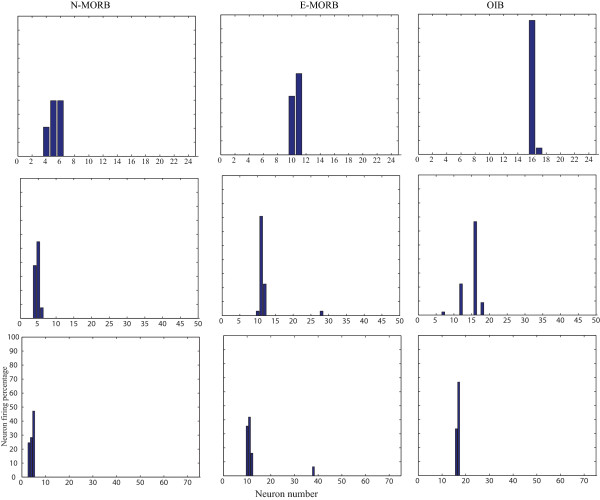
**Test result of LVQ after successful training of the architecture to classify N-MORB, E/P-MORB and OIB using 25, 50 and 75 neurons**. (a) The use of 25 neurons for identification of different basalts indicates that the network performed excellently and gives 100% satisfactory result for N-MORB, E/P-MORB and OIB. (b) A 50 neuron architecture shows a 100% satisfactory result for N-MORB whereas a mis-classification is noticeable for E/P-MORB and OIB. (c) A 75 neuron architecture performs 100% for N-MORB and OIB while a mis-classification occurs for the E/P-MORB.

Three different weight-matrixes of CVs were used for the three types of basalts. The initial weight-matrix of CVs was updated during training of the network and when the network reached its optimum efficiency the final weight-matrix was saved and used to classify the unknown data. In all the cases the network showed a satisfactory result by classifying the known data between 100% and 95%. Due to 100% classification of known N-MORB, E/P-MORB and OIB basalts data, a need did not arise to use the LVQ2 and LVQ3 architectures. LVQ1 architecture with 25 neurons performed very satisfactorily whereas with 50 and 75 neurons the possibility of mis-classification for E/P-MORB and OIB increased (Fig. 4). As it took less time for completion of the classification hence an architecture of 25 neurons was used (Table 1).

To help identify the involved characters in the data set, filters were designed using the optimized and final weight-matrix of the CVs. The filters are similar to the testing part of the LVQ1 architecture. While passing through the filters the network identifies the individual characters of the unknown data and this recognition is dependent upon the available characters of the basalts in the form of CVs in the weight-matrix.

## Classification Of Unknown Basalt Data

Sampling in the CIOB recovered a variety of rocks such as basalts, ferrobasalts, spilites and pumice clasts [23]. Basalts occur as pillows, large outcrops and as fragments. Compositionally, the basalts are Normal-MORB (N-MORB) similar to those from the Mid-Atlantic Ridge and East Pacific Rise [24]. Ferrobasalts, recovered near topographic highs and high amplitude magnetic zones, consist of plagioclase (predominant), sometimes olivine and frequently small euhedral magnetite and hematite grains [25]. Spilites, occurring near the Indrani fracture zone (79°E), show fine to medium grains of albitic plagioclase, clinopyroxene and olivine while epidote, hematite, chlorite and ore minerals form minor constituents. Pumices encompass a large field and are trachyandesite to rhyodacite in composition [26].

The CIOB basalts show considerable ranges in concentrations of the incompatible elements (e.g., Zr = 63-228 Xppm; Nb = 0.95-5 ppm; Ba = ~15-78 ppm; La = ~3-16 ppm) [27,28]. The incompatible elements (Ba, Zr, Nb, REE) with bulk distribution coefficients less than 1 (D<<1), show systematic enrichments with decreasing MgO where as the incompatible elements Sr and Sc (D ≥ 1), exhibit a scattered distribution. This could be accounted by the fractionation of olivine ± clinopyroxene and is also supported by the CaO/Al_2_O_3 _ratio of the samples.

**Figure 5:**
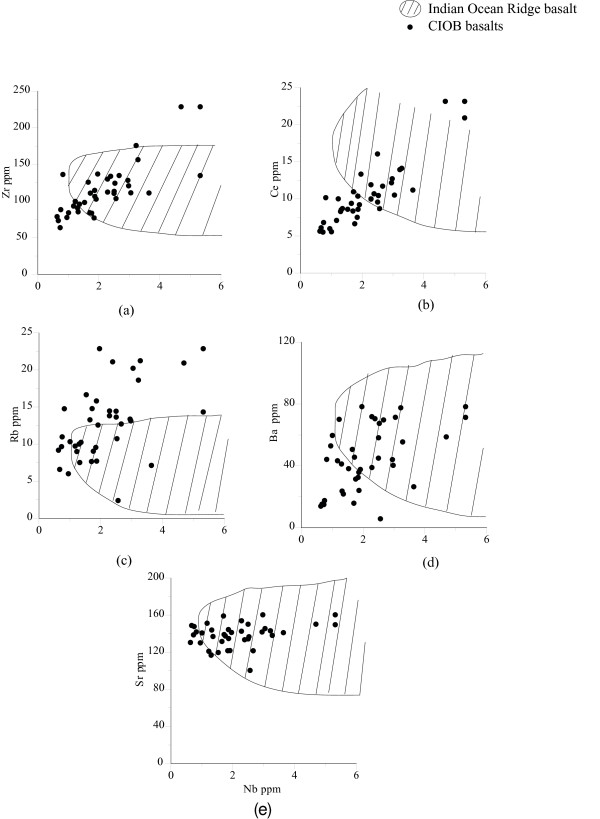
**The incompatible elements Zr, Ce, Rb, Ba and Sr show a variable distribution with Nb of the CIOB basalts**. The hatched area represents IOR basalts (data from PETDB http://www.petdb.org/). Note that Zr, Ce and Ba show a relative depletion whereas Rb shows relative enrichment than the IOR basalts for a given Nb concentration. Sr shows a very close association with the IOR basalts.

In general, the CIOB basalts have distinct incompatible element ratios e.g., Zr/Nb = 25-125, Y/Nb = 7-63 and (La/Sm)_N _= 0.5-1.5 [27,28]. The binary plots of Zr, Rb, Ce, Sr and Ba show a variable distribution against Nb (Fig. 5). Zr and Ce show a strong positive correlation with increasing Nb, whereas Rb and Ba show a scattered distribution and Sr shows a narrow trend. Observations indicate that when compared with N-MORB the CIOB basalts are relatively enriched in incompatible elements (Zr = 63-228 ppm, Y = 31-86 ppm) and relatively less incompatible element abundance than the K-P (K_2_O and P_2_O_5_) rich basalts from the Deep Sea Drilling Project (DSDP) Site 215, situated at the eastern margin of the basin [29,30]. An important aspect of the geochemical signatures of the CIOB basalts is the significant fractionation among the highly incompatible elements. For example, the Ba/La ratio is a factor of ~5 higher than typical N-MORB, where as the moderately incompatible element ratio such as Sm/La (0.4 to 1.1) is very close to the N-MORB [27,28].

**Figure 6:**
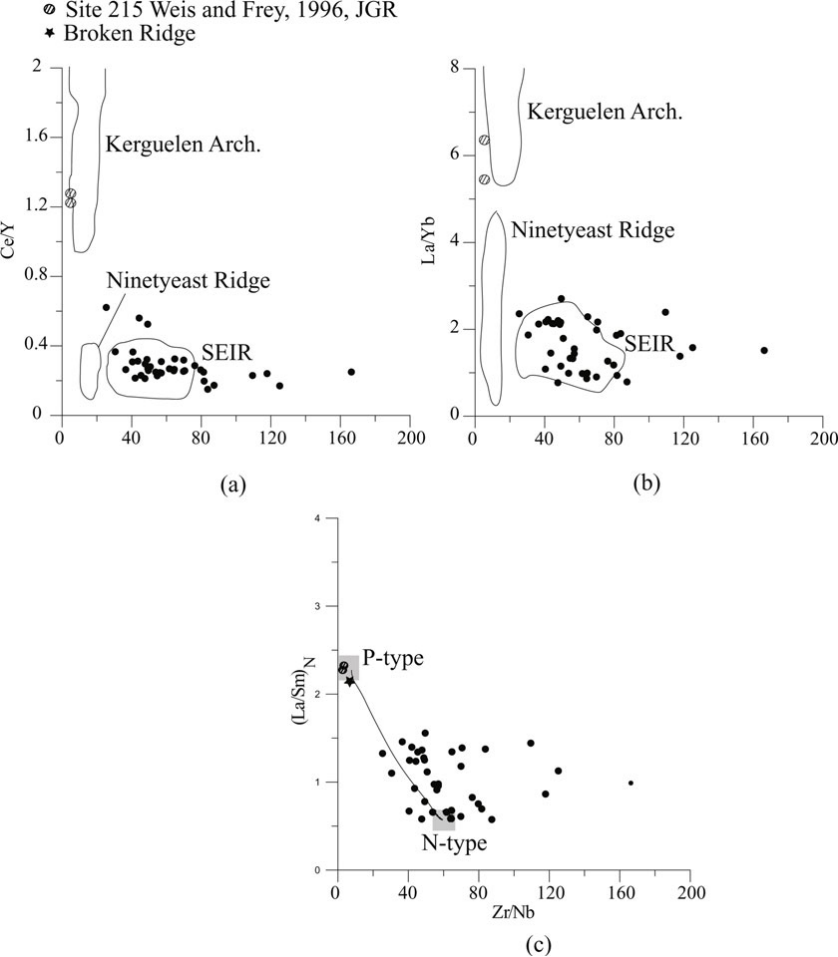
**(a) The variation of Ce/Y-Zr/Nb ratio of the CIOB basalts mostly falls in the SEIR domain and indicates a genetic relation**. In comparison, the basalts from Ninetyeast Ridge and Site 215 [30] do not show any relation with the CIOB basalts. (b) La/Yb-Zr/Nb ratios show a clustering and most of the data fall in the SEIR segment whereas Ninetyeast Ridge and Site 215 [30] again are not related with the CIOB basalts. (c) (La/Sm)N-Zr/Nb binary mixing diagram indicates that the CIOB basalts are mainly N-MORB with some component of E/P-MORB. For comparison data from Site 215 and Broken Ridge [29,30] have been plotted.

**Figure 7:**
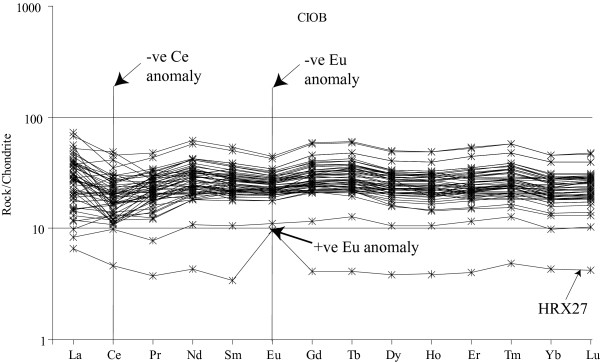
**The REE variation in the CIOB basalts indicates a moderate enrichment of LREE as compared to the N-MORB**. Representative N- and E-MORB variations are shown to indicate the enriched pattern of the CIOB basalts [28]. These patterns are quite similar to the LREE of the E/P-MORB where as the HREE variation is similar to N-MORB.

The Zr/Nb ratio serves as a useful information to identify the nature of the MORB. The CIOB basalts have high Zr/Nb (>25) [28] similar to typical N-MORB (> 30) [7]. The plots of Ce/Y vs Zr/Nb and La/Yb vs Zr/Nb indicate a close association of the CIOB basalts with the Southeast Indian Ridge (Fig. 6a, b). The plot (La/Sm)_N _vs Zr/Nb (Fig. 6c) indicates that although the CIOB basalts are typical N-MORB yet, faint signatures of E/P-type MORB are noticeable in the mixing relation between N- and P- types and this may be indicative of a low degree of partial melting of the source rock. The La/Yb and Ce/Y ratios (~0.7-2.7 and ~0.15-0.62, respectively) of the CIOB basalts are close to the chondrite values (La/Yb ≈ 1.39 and Ce/Y ≈ 0.39) [31] and indicate that these ratios were affected by the fractional crystallization of olivine and pyroxene. The chondrite normalized REE of the CIOB basalts also attest to the N-MORB nature of these basalts ([La/Yb]_N _of ~1.0) [28]. Interestingly, the CIOB basalts show enriched LREE and relatively flat HREE pattern (Fig. 7).

**Figure 8:**
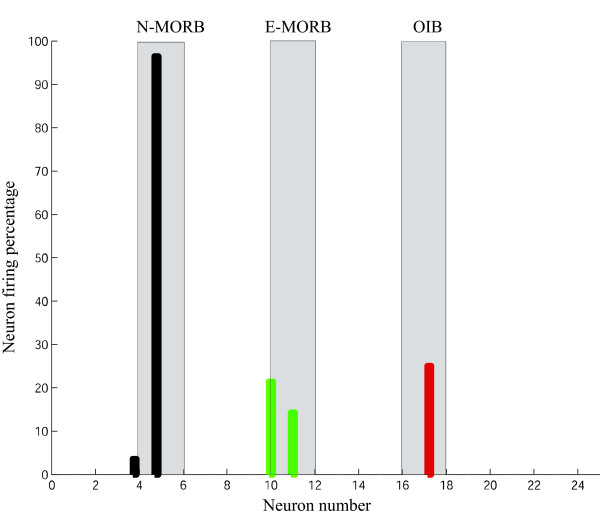
**A use of the ANN network with 25 neurons indicates the CIOB basalts to be dominantly N-MORB in nature but a few samples have a combination of either E/P-MORB or OIB character or E/P-MORB and OIB characters along with the N-MORB character**.

The LVQ analysis of the geochemical data of the CIOB basalts produced the following results (Fig. 8; Table 3):

**Table 3:** Characterisation of the CIOB basalts in terms of inherent character as N-MORB, E/P-MORB and OIB using LVQ (ANN architecture) technique.

Sample	N-MORB	E-MORB	OIB
PD1	√		
PD2	√	√	√
PD3	√		√
PD4	√		
PD5	√		√
PD6	√	√	√
PD7	√	√	√
PD8	√		
PD9	√		
PD10	√		
PD11	√		
PD12	√		
PD14	√		
PD15	√		
PD16	√		
PD17	√		
PD18	√		
PD19	√	√	√
PD20	√	√	
PD21	√		√
PD22	√		
PD25	√	√	
PD26	√		
PD27	√		
PD28	√	√	√
PD29	√		
PD30	√		
PD31	√		
PD32	√		
PD33	√		
PD35	√		
PD37	√	√	
HRX1	√		
HRX2	√		√
HRX3	√		√
HRX5	√	√	
HRX8	√		
HRX9	√		
HRX13	√	√	
HRX15	√		
HRX18	√		
HRX20	√		
HRX21	√	√	√
HRX22	√	√	
HRX27	√	√	
HRX28	√		
HRX29	√	√	
HRX30 (1)	√		
HRX31	√		
HRX34	√	√	
HRX35	√	√	√
HRX36	√	√	√
HRX37	√	√	
SS13-TS 90/91	√	√	
AAS22 DR#19	√	√	
SS11	√		

1) 57% of the basalts are typical N-MORB

2) 20% of the basalts have both N-MORB and E/P-MORB characters

3) 11% of the basalts show a combination of N-MORB and OIB signatures

4) 12% of the basalts have a mixed nature of N-MORB, E/P-MORB and OIB.

Thus, the CIOB basalts are largely N-MORB but in terms of certain elemental concentrations a few basalts have characteristics of the three basic groups of the OFB. This indicates the inhomogeneity of the source region together with variable melting of the source.

## Conclusion

It is well recognized that the geochemical study of basalts together with discrimination plots of selective elements and their ratios could help to identify the basic volcanics vis-è-vis their tectonic settings. The purpose of this work however, was to highlight the development of a suitable real-time program to help classify the oceanic basalts on the basis of their discrete geochemical characters which may not be fully revealed in the classical discrimination diagrams. In this respect, the need of soft computational techniques (like ANN) is useful and faster.

The present study indicates that the supervised LVQ1 architecture performs satisfactorily to identify the geochemical characters in the data and the possibility of mis-characterization is minimal. Further work could help to refine the model by a possible reduction in the number of variables that are needed for the classification scheme.

## Competing interests

The authors declare that they have no competing interests.

## Authors' contributions

PD was responsible in developing the idea, computation of the data and writing of the first version of the manuscript. SDI helped to further, discuss the processes involved and in revising the manuscript. The sampling and data collection were conducted by both the authors. All authors read and approved the final manuscript.
